# Exacerbation of Galli-Galli Disease Following Dialysis Treatment: A Case Report and Review of Aggravating Factors

**DOI:** 10.7759/cureus.15401

**Published:** 2021-06-02

**Authors:** Tejas P Joshi, Sally Shaver, Jaime Tschen

**Affiliations:** 1 Dermatology, Baylor College of Medicine, Houston, USA; 2 Dermatology, Conroe Dermatology Associates, Conroe, USA; 3 Dermatology, St. Joseph Dermatopathology, Houston, USA

**Keywords:** end-stage renal disease, galli-galli disease, dowling-degos disease, genodermatosis, dialysis, contact dermatitis

## Abstract

Galli-Galli disease (GGD) is a rare genodermatosis that is an acantholytic variant of Dowling-Degos disease that presents as lentigo-like macules/papules with progressive reticulated hyperpigmentation. Heat, sweat, ultraviolet light exposure, and topical retinoids have been reported to exacerbate the lesions associated with GGD. Here, we present a 77-year-old woman with end-stage renal disease and GGD who reported a worsening of lesions during the summer months and following hemodialysis treatment. Despite the severity of her lesions following dialysis, she refused treatment with isotretinoin out of concern for its side effect profile. In this case report, we discuss some available treatment options for GGD and review the exacerbating factors for GGD currently reported in the literature.

## Introduction

Galli-Galli disease (GGD) is a rare, autosomal dominant genodermatosis that is an acantholytic variant of Dowling-Degos disease (DDD). DDD encompasses a spectrum of skin conditions that present with progressive reticulated hyperpigmentation; while GGD was initially postulated to be a distinct entity from DDD, more recent evidence has led to the consensus that GGD is a variant of DDD [1]. Clinically, GGD is indistinguishable from classic DDD and typically involves the flexural areas and presents as erythematous, pruritic, scaly lentigo-like macules/papules with progressive reticulated hyperpigmentation [2]. Histologically, acantholysis is considered to be a *sine qua non* for the diagnosis of GGD [2] and is often accompanied by hyperkeratosis, dermal fibrosis, elongated rete ridges, and infiltrating lymphocytes [3]. Mutations in *keratin 5* (*KRT5*) [4,5] and *protein O-glucosyltransferase 1* (*POGLUT1*) [6] have been identified in patients with GGD. GGD does not appear to have a predilection for any particular race or gender [1]. There is currently no established paradigm of care for GGD, but topical retinoids [7] and laser treatment [8,9] have been reported to be efficacious treatments in the management of GGD.

## Case presentation

**Figure 1:**
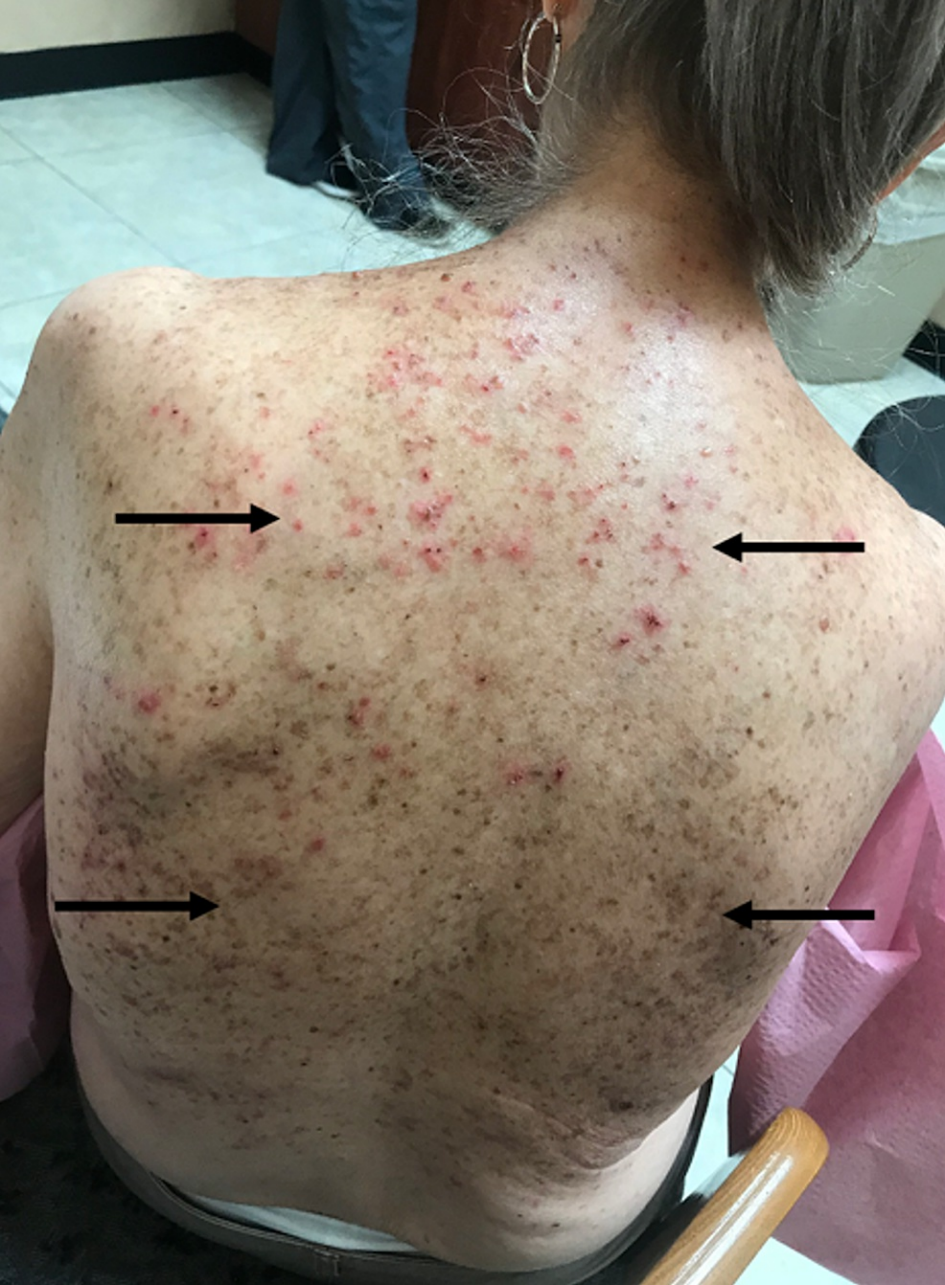
Papules/plaques present on the patient’s back. The bottom two arrows point to hyperpigmented lesions characteristically observed in Galli-Galli disease.

A 77-year-old woman with end-stage renal disease (ESRD) requiring hemodialysis presented for evaluation of pruritic papules and plaques on her neck, back, and chest (Figure 1). The patient mentioned that she had papules/plaques in these regions for many years and that they worsened during the summer months. She also mentioned that the lesions flared up considerably after her dialysis visits, causing intense pruritus. The patient had long-standing hypertension which had gone undiagnosed and had precipitated her ESRD; she had been on dialysis for 4.5 years. Her family history was negative for GGD.

**Figure 2:**
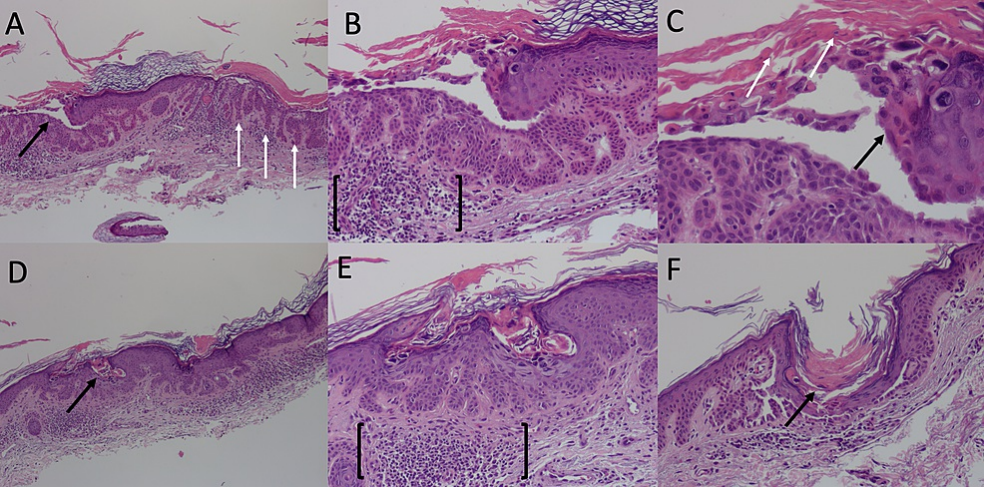
Histopathology of shave biopsy of the skin over the right mid back and over the right chest. (A) Black arrow points to the area of focal acantholysis; white arrows point to elongated filiform rete ridges, 40× magnification; (B) brackets outline region of lymphocytic infiltration, 100× magnification; (C) black arrow points towards the region of dyskeratosis and white arrows point to parakeratosis, 200× magnification. (D) Black arrow points to the area of focal acantholysis, 40× magnification; (E) brackets outline the region of lymphocytic infiltration, 100× magnification; (F) arrow points to the region of acantholysis, 200× magnification.

A 0.7 cm × 0.5 cm shave biopsy of the skin over the right mid back and a 0.7 cm × 0.6 cm shave biopsy of the skin over the right chest were taken. Histopathologic findings for both biopsies were similar and revealed a small focus of acantholytic dyskeratosis accompanied by suprabasal acantholysis, both features suggestive of a differential diagnosis of Darier’s disease, GGD, Hailey-Hailey disease, or transient acantholytic dermatosis (Grover’s disease). However, the lentiginous background, together with the elongated filiform rete edges, clinches the diagnosis of GGD. Dyskeratosis, parakeratosis, and lymphocyte infiltration were also noted on biopsy (Figure 2).

Our patient was treated with prednisone, which offered some relief of pruritic symptoms, yet did not induce any regression of lesions. She was offered isotretinoin therapy but declined due to concerns regarding its side effect profile.

## Discussion

GGD was first described in 1982 by Bardach et al. in two brothers who would become eponymous for the disease. These brothers presented with hyperpigmented macules distributed over the face, neck, and flexural areas. Histopathologic examination showed acantholysis and formation of lacunae. Inspection of the biopsy under electron microscopy revealed separation of desmosomes and thickened collagen-like tonofilaments. Both brothers reported their lesions to be aggravated by heat [10].

Since the 1982 publication of the first reported cases of GGD, further studies have helped elucidate the genetics underlying GGD. GGD is an autosomal dominant genodermatosis that typically presents in the fourth decade [1]. A complete genetic picture of GGD is yet to emerge, but pathogenic variants in *KRT5* [4,5] and *POGLUT1 *[6] have been identified as culprits for GGD. The precise pathogenesis of GGD also remains poorly understood, but in the case of *KRT5* variants, it is likely that an aberrant keratin 5 polypeptide compromises stratum basale integrity [2], and in the case of *POGLUT1* mutation, it is likely that an abnormal O-fucosyltransferase 1 transcript impairs Notch signaling, a critical pathway for melanocyte and keratinocyte proliferation and differentiation [11]. However, as was the case in our patient, GGD can also arise sporadically [1].

GGD can present similarly to Grover’s disease, which is a related genodermatosis presenting as a papulovesicular rash over the trunk. Histologically, Grover’s disease is also similar to GGD and presents with acantholysis and dyskeratosis. The two diseases are also exacerbated by similar triggers [12]. Given the similarities in the presentation of both diseases, it is possible to suggest Grover’s disease as an alternative diagnosis for our patient; however, we argue that our patient’s presentation is more consistent with GGD rather than Grover’s disease.

Indeed, the truncal distribution of lesions in our patient is atypical for a presentation of GGD, which classically involves the flexural areas. However, 15 cases of GGD sparing the flexural areas have been reported in the literature [1]. Moreover, the elongation of rete ridges observed on histopathologic examination (Figure 2A) is not observed in Grover’s disease and is more consistent with a diagnosis of GGD; furthermore, the basal layer hyperpigmentation that is observed on histopathologic examination is a hallmark of GGD, yet an atypical finding in Grover’s disease [12]. The lack of relevant family history in our patient might also seem unusual. However, 26 spontaneous cases of GGD have been noted in the literature [1]. Altogether, we note that the morphological and histological features of our patient’s lesions are consistent with GGD. We acknowledge, however, the lack of a genetic diagnosis in our patient to be a limitation of our case report.

GGD tends to present with pruritus, although not all patients with GGD experience pruritus [1]. In the literature there are no cases that thoroughly assess the quality of life of patients with GGD, although it is likely that from a cosmetic standpoint, the lesions are irritating; the associated pruritus may also impair the quality of life of patients with GGD. Our patient reported the pruritus associated with her GGD to be “relentless,” especially following dialysis treatment, and noted that it significantly impaired her quality of life.

There is currently no established paradigm of care for GGD and there is a paucity of evidence to definitively establish the efficacy of any particular treatment. Some cases of GGD have shown response to topical retinoids [7], yet cases of GGD refractory to acitretin have also been reported in the literature [13]. Furthermore, the side effects of topical retinoids have been well described in the literature [14], and our patient declined treatment with isotretinoin due to concerns over its side effect profile. We summarize some of the common cutaneous and systemic side effects of isotretinoin therapy in Table 1 [14].

**Table 1:** Common cutaneous and systemic side effects of isotretinoin [14]

Cutaneous side effects	Systemic side effects
Facial erythema	Arthralgia
Pruritus	Headache
Retinoid dermatitis	Myalgia
Skin exfoliation	Suicidal ideation
Xerosis	Tiredness

Mota et al. reported topical calcineurin inhibitors to be initially successful in a patient with GGD, yet continued treatment was ineffective [15]. GGD has also been reported to be generally recalcitrant to topical and systemic steroid treatment [1].

There is, however, a modicum of evidence to support the use of lasers. Voth et al. reported a case of erbium-doped yttrium aluminum garnet (Er:YAG) laser leading to complete resolution of lesions [8]. Additionally, Venning et al. showed Er:YAG laser, intense pulse light, and electrofulguration to be all equally efficacious in the management of GGD [9].

We also note that the treatments for GGD are similar to the treatments for Grover’s disease: corticosteroids and retinoids often constitute the first-line treatment for both conditions [1,12]. Given the similarity of treatment options for GGD, we are led to speculate that novel therapies used for Grover’s disease may also have an application in the management of GGD. Of note, we speculate that perhaps dupilumab and naltrexone may aid in the management of patients with GGD. Dupilumab has been reported to lead to remarkable control of Grover’s disease in three patients. Importantly, all of the patients tolerated dupilumab well, suggesting that it may be both an efficacious and safe treatment for Grover’s disease, and perhaps by extension, other closely related genodermatoses such as GGD [16]. Naltrexone has also shown some promise in the management of Grover’s disease and may represent another therapeutic alternative for patients with GGD [17].

A number of factors have been reported to exacerbate GGD, with heat being the most common offending agent. Both brothers who were the first cases of GGD reported their GGD to be worsened by heat [10]. In a case series of 18 patients, Hanneken et al. reported that nine patients experienced worsening of their GGD due to heat [18]. In their case series, Hanneken et al. also identified sweat and ultraviolet (UV) light to be common aggravating factors, with 10/18 patients reporting worsening of their lesions due to sweat and 7/18 patients reporting worsening of their lesions due to UV exposure [18].

The triad of heat, sweat, and UV light have been all implicated in the exacerbation of GGD in four other patients [1,2,15,19]. In one patient, it was actually winter that exacerbated GGD [20]. Interestingly, topical retinoids (which, as discussed above, have shown some promise in the management of GGD [7]) have also been reported to in fact worsen GGD [4].

Our patient reported her GGD to worsen during the summer months, and this is consistent with the pattern of GGD exacerbation due to heat that is well-described in the literature. However, our patient also reported worsening of her GGD lesions and a marked increase in pruritus following dialysis treatment. To our knowledge, this is the first case report which describes the flare-up of GGD following dialysis treatment.

## Conclusions

GGD is a rare, autosomal dominant genodermatosis that manifests as erythematous, hyperpigmented macules/papules and presents typically on the flexural areas. Histologically, it is characterized by acantholysis, hyperkeratosis, elongated rete ridges, and lymphocyte infiltration. A complete genetic picture of GGD is yet to emerge, although mutations in *KRT5* and *POGLUT1* have been identified as culprits for GGD. Management of GGD is challenging, and although topical retinoids have shown some promise, not all cases of GGD are responsive to topical retinoid treatment, and some cases of GGD in fact exacerbate with treatment. Additionally, as was the case in our patient, individuals may reject topical retinoid treatment due to concerns over side effects. GGD is most often exacerbated by heat (as seen in our patient), sweat, UV exposure, as well as isotretinoin treatment. Our patient also reported worsening of her lesions following dialysis treatment, and to our knowledge, represents the first report of a patient whose GGD flared following dialysis treatment.
